# Fast Optical Coherence Elastography via Optimized Sampling Density With Preserved Accuracy

**DOI:** 10.1002/jbio.70376

**Published:** 2026-10-19

**Authors:** Guangyu Zhang, Zhengshuyi Feng, Alison M. Layton, Chunhui Li, Zhihong Huang

**Affiliations:** ^1^ Healthcare Engineering, School of Physics and Engineering Technology University of York York UK; ^2^ Hull York Medical School University of York York UK; ^3^ Biomedical Engineering, School of Science and Engineering University of Dundee Dundee UK

## Abstract

Optical coherence elastography (OCE) is a non‐invasive imaging technique for quantifying tissue stiffness. The spatial resolution of elastography maps is closely related to the spatial sampling density required to adequately represent mechanical features. In this study, we investigated the effect of spatial sampling density on OCE by varying the number of effective sampling points along scanning axes using the proposed phase‐gradient velocity estimation method. These methods were validated using agar phantoms and in vivo human facial acne and the results showed that down sampling reduced computational cost by around 69% and up to about 89%. For the down sampling results of the acne dataset, the velocity changed by only 5.82% in an affected lesion. These results provide a practical criterion for reducing acquisition and post‐processing time while establishing the quantitative relationship between spatial sampling, elastography accuracy and computational efficiency.

## Graphical abstract

In this study, we investigated the effect of spatial sampling density on OCE by varying the number of effective sampling points along the scanning axes using the proposed phase‐gradient velocity estimation method.

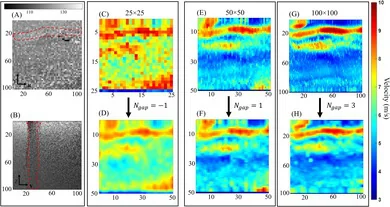

## 1 Introduction

Elastography is a non‐invasive medical technique used to assess tissue stiffness by estimating mechanical properties such as Young's modulus [1, 2, 3]. Among medical imaging modalities, ultrasound elastography [4, 5], magnetic resonance elastography (MRE) [6, 7] and optical coherence elastography (OCE) [8, 9, 10] are widely applied to quantify the mechanical properties of tissue in dermatology [2]. OCE relies on optical coherence tomography (OCT) to capture wave propagation by external stimulation and reconstruct high spatial resolution elastography in superficial tissues. However, achieving high spatial resolution in OCE inevitably requires longer acquisition times and increases the post‐processing burden. Long acquisition duration may induce motion artefacts caused by involuntary human movement [11, 12, 13], which would influence the accuracy of wave velocity estimation. Improving efficiency while maintaining image quality is therefore essential to support the broader adoption of elastography in dermatology [14] and ophthalmology [15]. Several approaches have been applied to accelerate OCE acquisition by hardware improvement, scanning protocol and deep learning. Singh et al. proposed line‐field OCE using a Michelson‐type interferometer to achieve rapid imaging of elastic waves [16]. Lu et al. developed swept‐source scanning OCE (SSS‐OCE) to accelerate the acquisition, where a dispersive element generates a lateral line scan and temporal information is constructed from multiple line scans [17]. While these approaches significantly enhance imaging speed and reduce acquisition time, the reduced temporal sampling density may limit depth‐resolved accuracy and affect the fidelity of wave propagation tracking. In addition to these previous approaches, Neidhardt et al. presented a miniaturized fibre‐scanning endoscope combined with deep learning to reconstruct three‐dimensional (3D) stiffness maps [18]. However, the conical scanning pattern has the potential to lead to insufficient sampling density in the lateral or depth directions.

The spatial resolution of OCE is fundamentally inherited from the underlying OCT system [19]. However, the effective spatial resolution of elastography does not directly equal the structural resolution of OCT [20]. It is constrained by the mechanical excitation characteristics, such as the pulse width and resulting wavelength of the propagating wave, which determine the smallest resolvable mechanical feature. Furthermore, the sampling density used during data acquisition also influences the effective resolution of the elastography map. Apart from the physical spatial resolution, the computational strategy used for velocity estimation further influences the effective elastography resolution of the reconstructed stiffness map [21]. Different estimation approaches introduce distinct spatial smoothing or fitting effects, thereby modifying the effective spatial resolving capability. Therefore, the effective elastography resolution should be considered as a combined outcome of OCT system resolution, excitation pulse characteristics, sampling density and the adopted velocity estimation algorithm.

Traditional velocity estimation methods such as time‐of‐flight (TOF) based on cross‐correlation present accuracy and efficiency limitations [22, 23]. In TOF, a reference point is selected and the time lag corresponding to the maximum cross‐correlation between the reference and neighbouring points is calculated. The local velocity is then estimated by dividing spatial distance by the measured time delay within a moving window [24]. However, this method is sensitive to reference point selection; in addition, subjective choice may lead to inconsistencies in stiffness allocation. Moreover, cross‐correlation requires repeated domain transformations between time and frequency, increasing computational load and post‐processing time. However, this problem can be resolved by utilizing phase‐gradient velocity estimation, a method already established in ultrasound elastography [23].

To establish a quantitative framework for optimizing spatial sampling in OCE, this study develops sampling strategies along both the fast and slow scanning axes based on the proposed phase‐gradient velocity estimation method. This work provides practical guidance for OCE system design and acquisition optimization by jointly considering OCT resolution, surface acoustic wave (SAW) wavelength, spatial sampling density and the underlying velocity estimation algorithm. The framework establishes quantitative sampling criteria that characterize the trade‐off between elastography accuracy and acquisition efficiency.

## 2 Methods

### 2.1 Scanning Protocol

In the scanning protocol, the laser beam scans continuously along the lateral direction to define the scanning region. For each excitation event, a two‐dimensional (2D) spatial OCE dataset is acquired with dimensions of $N_{Z}\times N_{Y}\times N_{X}$, $N_{Z}$ denotes the number of axial pixels (384 pixels in this study), $N_{Y}$ represents the number of lateral sampling positions along the fast axis and $N_{X}$ corresponds to the number of A‐lines acquired over time (600 pixels in this study). The resulting 3D spatial dataset has dimensions $N_{Z}\times N_{Y}\times N_{R}\times N_{X}$, where $N_{R}$ denotes the number of repeated B‐scans along the slow axis.

In high‐resolution (HR) sampling mode, all A‐lines are sequentially acquired, forming complete B‐frames and repetition frames in Figure 1. In contrast, low‐resolution (LR) mode skips adjacent sampling points according to a predefined interval while preserving the overall spatial indexing structure. The parameter $N_{\mathrm{gap}}$ denotes the sampling gap between neighbouring acquired points and the scanning mode will be defined in Section 2.3.

**FIGURE 1:**
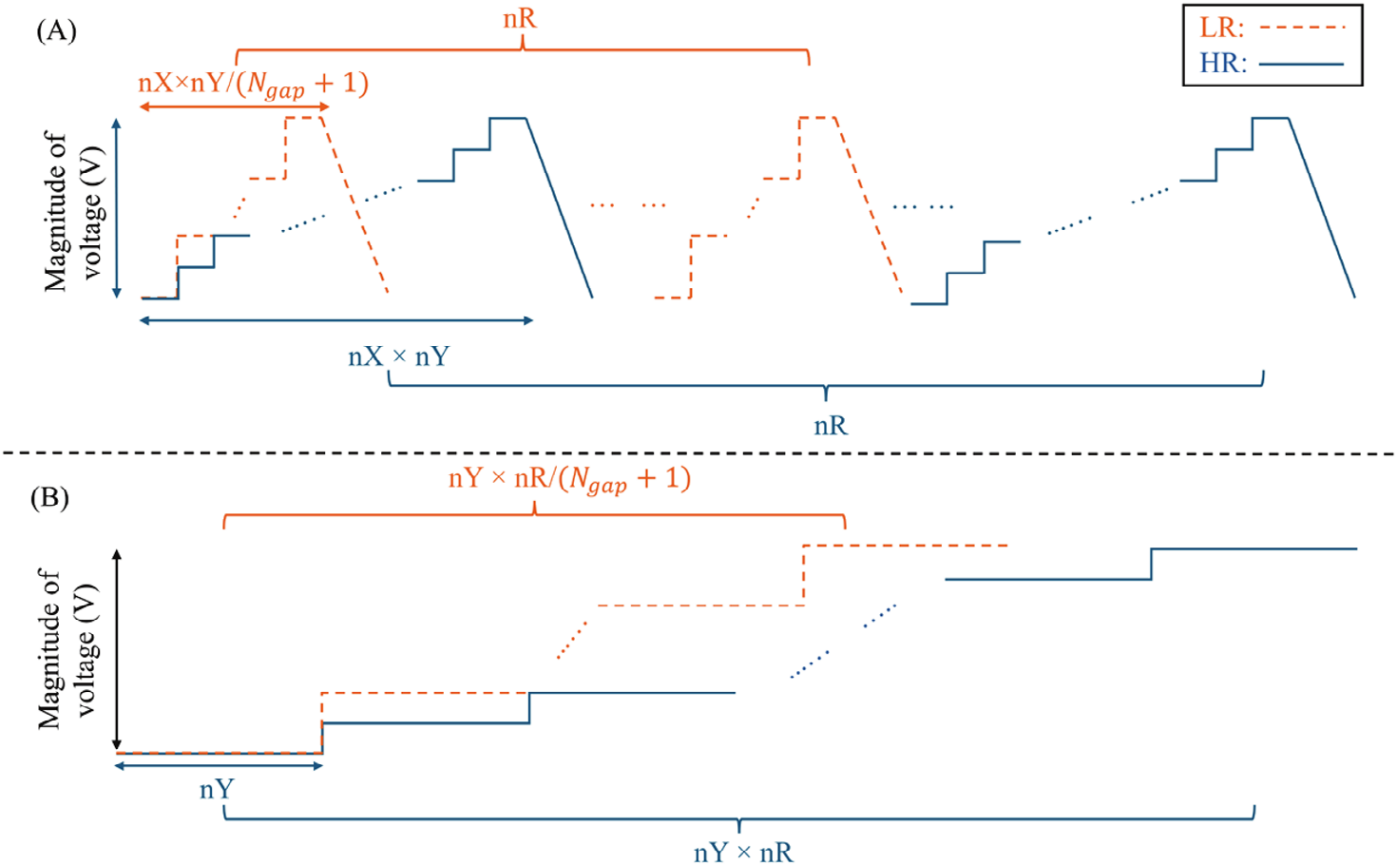
The schematic illustration of (A) is the lateral scanning direction and (B) is the repetition scanning direction.

### 2.2 Relationship Between Spatial Resolution in OCT/OCE and SAW Wavelength

This section defines and analyses the lateral resolution of the single B scan in OCT acquisition ($R_{\mathrm{OCT}}$), the SAW wavelength (*λ*) and the effective resolution of OCE mapping ($R_{\mathrm{OCE}}$).

#### 2.2.1 OCT Effective Lateral Sampling Resolution

The experimental lateral resolution of a single B scan in air is determined by the spatial sampling interval along the fast‐scanning axis. It depends on the relationship between total lateral scanning range ($R_{S}$) and number of sampling points ($P_{S}$) and the effective $R_{\mathrm{OCT}}$ is defined as:

(1) $$R_{\mathrm{OCT}}=\frac{R_{S}}{P_{S}}$$

#### 2.2.2 OCE Mapping Resolution

The $R_{\mathrm{OCE}}$ is determined by the system point spread function (PSF), which reflects both OCT imaging resolution and the smoothing effect introduced by phase velocity estimation windows [19]. Assuming a Gaussian‐shaped PSF, the $R_{\mathrm{OCE}}$ is assumed as follows:

(2) $$R_{\mathrm{OCE}}=2.355\sqrt{\sigma_{\mathrm{OCT}}^{2}+\sigma_{\text{window}}^{2}}$$

where $\sigma_{\mathrm{OCT}}$ represents the standard deviation of $R_{\mathrm{OCT}}$ and $\sigma_{\text{window}}$ denotes the standard deviation of the velocity estimation window.

Since both the OCT effective sampling resolution and the velocity estimation window size (*W*) follow a rectangular distribution, the effective OCE resolution becomes:

(3) $$R_{\mathrm{OCE}}=0.68R_{\mathrm{OCT}}\sqrt{1+W^{2}}$$

### 2.3 Relationship Between $R_{\mathrm{OCE}}$ and Sampling Density

#### 2.3.1 OCE Effective Resolution Criterion After Down/Up Sampling

According to the Nyquist sampling theorem, the minimum resolvable feature must be at least twice the effective sampling interval. The smallest detectable wavelength at inclusion is constrained by:

(4) $$\lambda >2R_{\mathrm{OCE}}=1.36R_{\mathrm{OCT}}\sqrt{1+W^{2}}$$

To explore the effects of spatial sampling density on the OCE map. A $N_{\mathrm{gap}}$ represents the number of omitted ($N_{\mathrm{gap}}>0$) or inserted ($N_{\mathrm{gap}}<0$) points between adjacent captured samples:

(5) $$\lambda >1.36\cdot \left(\left|N_{\mathrm{gap}}\right|+1\right)\cdot R_{\mathrm{OCT}}\sqrt{1+W^{2}}$$

### 2.4 Phase Gradient‐Based Velocity Estimation

#### 2.4.1 Main Frequency Identification

A 4 kHz square‐wave excitation is employed in this study. Accordingly, the analysis frequency range is defined between 500 and 5000 Hz to encompass the dominant spectral components. For each depth, a fast Fourier transform (FFT), denoted as $F_{t}$, is applied along the temporal dimension to the phase signal $\phi \left(x,t\right)$, thus, the dominant frequency (*f*_m_) is determined based on the frequency corresponding to the maximum value of the average amplitude spectrum $\left(P\left(f\right)\right)$ across lateral positions within the predefined frequency range:

(6) $$P\left(f\right)=\frac{1}{N_{x}}\sum_{1}^{N_{x}}\left|F_{t}\left\{\phi \left(x,t\right)\right\}\right|$$

#### 2.4.2 Extraction of Lateral Phase Profile

At the identified main frequency $f_{m}$, the complex spectral component is extracted and then the lateral phase distribution is obtained from its argument. This unwrapped phase $\theta \left(x\right)$ represents the spatial phase profile of the propagating SAW at depth z:

(7) $$\theta \left(x\right)=\text{unwrap}\left(∠\phi_{f}\left(x,f_{m}\right)\right)$$

where $∠\left(\cdot \right)$ denotes the phase angle and phase unwrapping is performed along the lateral direction to ensure spatial continuity.

#### 2.4.3 Local Phase‐Gradient Estimation

To estimate the local wavenumber (k), a lateral sliding window is applied along the lateral direction at each depth. Within each window, the unwrapped phase samples are fitted using linear regression:

(8) $$\begin{array}{c} k=\frac{\mathrm{d\theta}}{\mathrm{dx}}=\frac{W\sum x_{i}\theta_{i}-\sum x_{i}\sum \theta_{i}}{W\sum x_{i}^{2}-{\left(\sum x_{i}\right)}^{2}},\;i=1,\ldots ,w \end{array}$$

where $x_{i}$ is the lateral coordinate of the *i*th pixel within the window and $\theta_{i}$ is the corresponding unwrapped phase value.

#### 2.4.4 Phase Velocity Calculation

The local phase velocity $\left(v\right)$ is determined from the dispersion relationship governing mechanical wave propagation. Velocity estimation was restricted to the top 100 pixels beneath the sample surface, where the signal‐to‐noise ratio (SNR) was sufficiently high to ensure reliable phase analysis. The local phase velocity is calculated using the dispersion relation:

(9) $$v\left(x\right)=\frac{2\pi f_{m}}{k\left(x\right)}$$

### 2.5 Quantification Metric

To evaluate the influence of up sampling and down sampling on effective resolution in OCE maps, a quantitative contrast analysis was performed based on the stiffness difference between inclusion and base regions. Maximum intensity projection (MIP) along the depth direction was first applied to generate en face elastography maps. The contrast‐to‐noise ratio (CNR) was then calculated to quantify the distinguishability between the inclusion and base regions as follows. A higher CNR value indicates stronger mechanical contrast and clearer boundary delineation, reflecting better effective elastography resolution.

(10) $$\mathrm{CNR}=\frac{|\mu_{\mathrm{inc}}-\mu_{\mathrm{bas}}|}{\sqrt{\sigma_{\mathrm{inc}}^{2}+\sigma_{\mathrm{bas}}^{2}}}$$

where μ and σ represent the mean and standard deviation of phase velocity within the inclusion (inc) and base (bas) regions, respectively.

To quantify boundary preservation after down sampling or up sampling, the boundary localization error was evaluated using the mean absolute error (MAE). Corresponding boundaries were manually delineated on the reference structural image and the boundary in the elastography was identified using the velocity gradient. The boundary localization error was then computed as the mean absolute vertical displacement between corresponding boundaries.

(11) $$\mathrm{MAE}=\frac{1}{N}\left|y_{1}\left(i\right)-y_{2}\left(i\right)\right|$$

where $y_{1}\left(i\right)$ and $y_{2}\left(i\right)$ represent the boundary positions in the reference image and the mapped down‐/up‐sampled image, respectively, at the *i*th lateral sampling location and N denotes the total number of lateral sampling points.

## 3 Materials

For the 2D OCE experiments, two configurations were prepared to eliminate directional bias, where the left and right regions were alternated between 1% and 2% concentrations. The agar fabrication process involved pouring one concentration into a mould to half volume and allowing it to cool to room temperature before adding the second concentration layer. Different $N_{\mathrm{gap}}$ values (6 and 69) were applied to investigate the influence of sampling density on phase velocity distribution and boundary sharpness. Up sampling was not implemented for 2D OCE, as the lateral resolution of acquired samples was already sufficiently high to resolve the boundaries in the agar sample.

For the 3D OCE experiments, inclusion phantoms were constructed to assess the ability of the proposed method to distinguish small boundary variations and phase velocity differences under different effective resolutions. The 1.5% agar base was first prepared and allowed to solidify; subsequently, 2.5% agar inclusion was injected using a 19G needle with an outer diameter of 1.1 mm. In addition, one volunteer with facial acne was recruited for the OCE study under the approval of the Physical Sciences Ethics Committee (PSEC) of the University of York [25]. All procedures conformed to the tenets of the Declaration of Helsinki. Volumetric datasets with lateral–elevational resolutions of 25 × 25, 50 × 50 and 100 × 100 (nY × nR) were acquired, all containing 384 pixels in the depth direction. The up sampling was applied to the 25 × 25 and down sampling was employed to the 50 × 50 and 100 × 100 datasets with designed $N_{\mathrm{gap}}$ values, respectively, −1, 1 and 3.

## 4 Experimental Setup

In this study, the OCE experimental system comprises two primary components, as illustrated in Figure 2: a swept‐source OCT (SS‐OCT) imaging module and a SAW excitation module [26, 27]. The SS‐OCT system operates at a sweep rate of 400 kHz, with a central wavelength of 1300 nm and a bandwidth of 100 nm. It provides a penetration depth of approximately 2 mm and an axial resolution of ~7.5 μm, while the lateral distance is around 5.1 mm and the corresponding resolution is ~8.6 μm in air. The scanning distance and resolution in the elevational direction are the same as those in the lateral direction. To capture SAW propagation within a 2D cross‐sectional region, an M–B scanning protocol was adopted.

**FIGURE 2:**
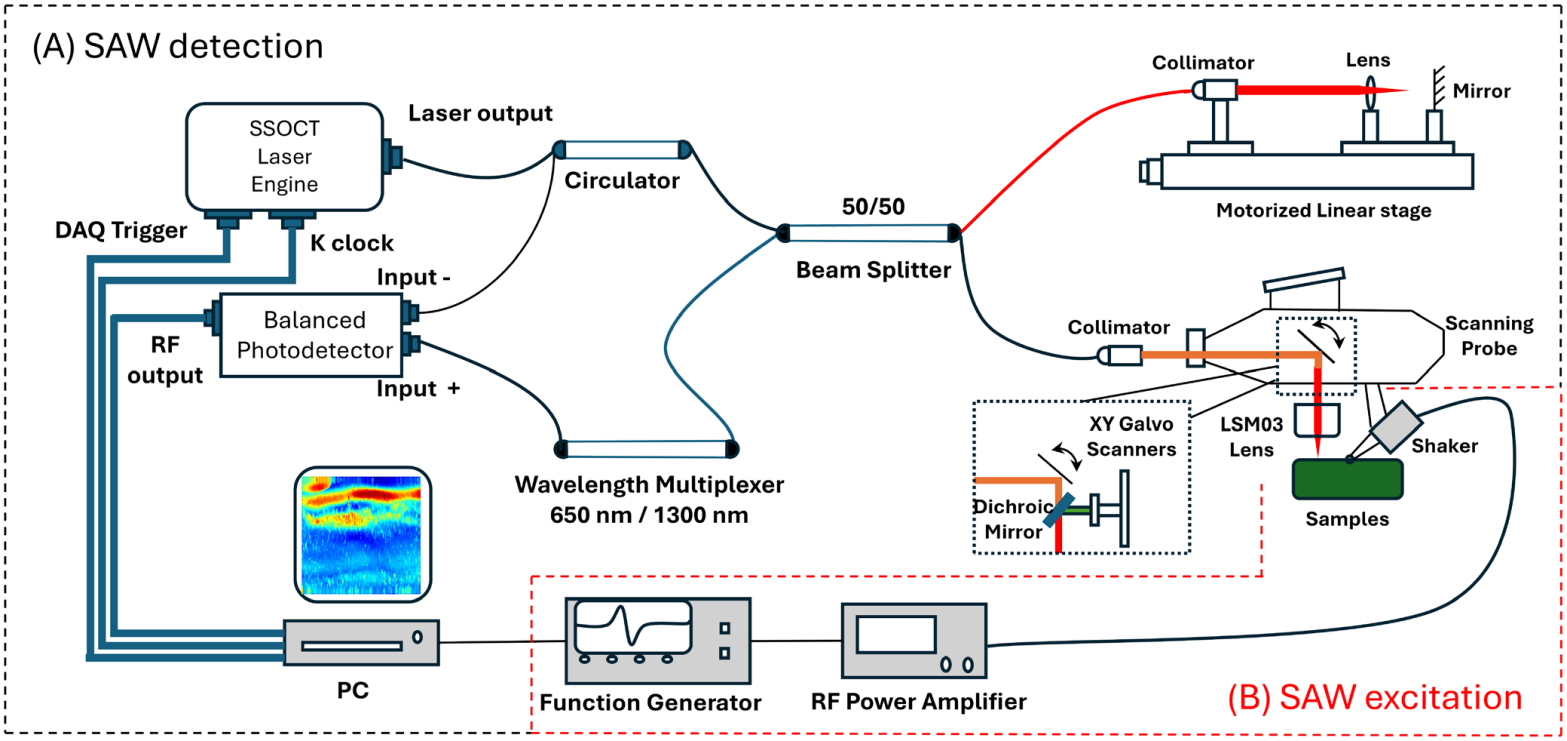
Schematic of the optical coherence elastography (OCE) experimental setup, consisting of (A) SAW detection based on swept‐source OCT and (B) SAW excitation using a contact‐based piezoelectric shaker.

## 5 Results

### 5.1 2D OCE Results

#### 5.1.1 Influence of Sampling Density on Velocity Estimation

For 2D OCE experiments, the mechanical stimulator was positioned on the left side of the sample in Figure 3. Figure 3A,B show the velocity maps obtained without down sampling for agar phantoms I and II, respectively. Figure 3C,D present the corresponding velocity maps acquired using the down‐sampling strategy with a sampling gap of 9, while the Figure 3E,F correspond to a more aggressive down‐sampling case with a gap of 69.

**FIGURE 3:**
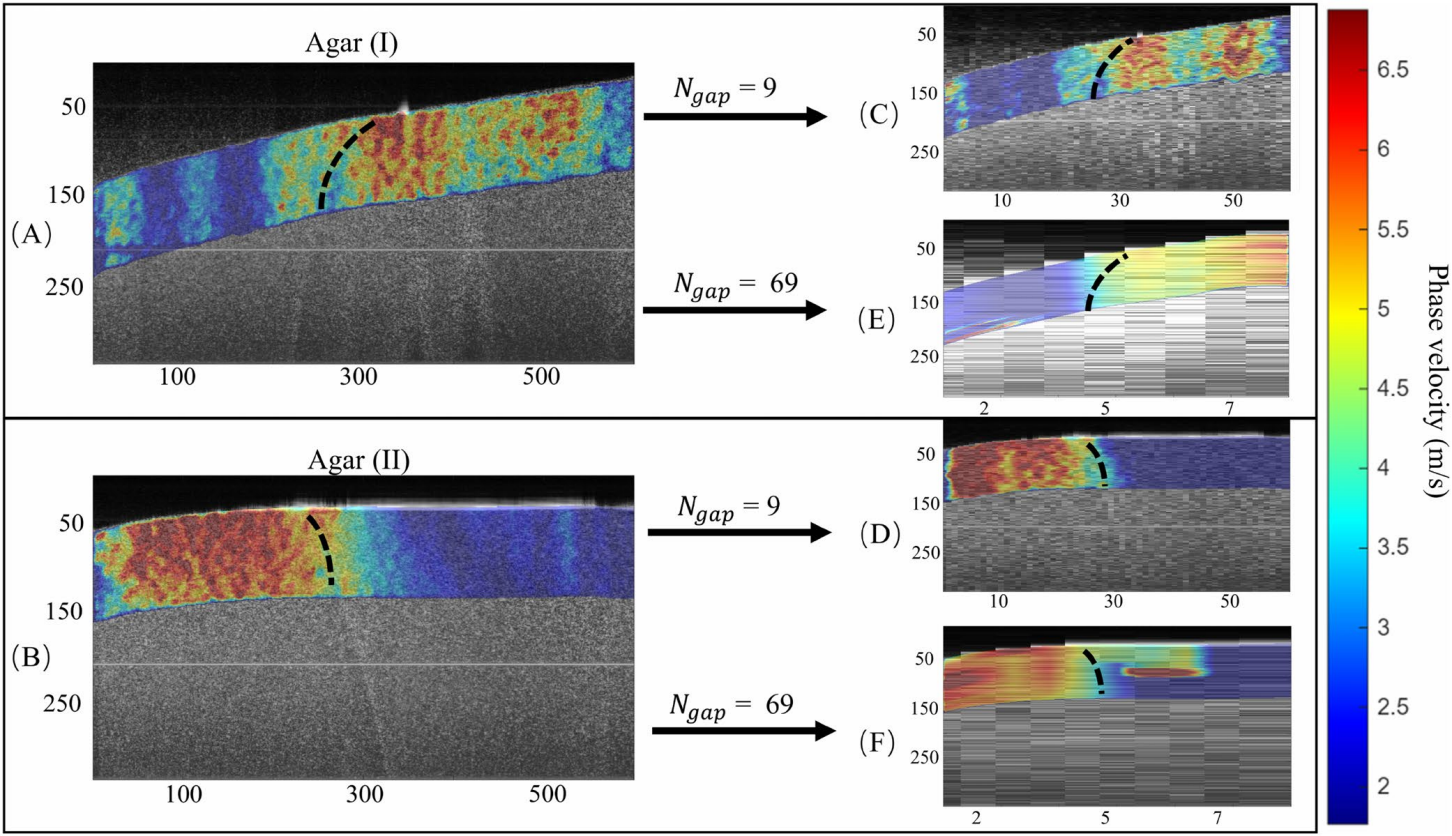
Two‐dimensional cross B‐scan OCE velocity maps of lateral‐difference agar phantoms. The top row corresponds to agar phantom (I), composed of 1% and 2% agar from left to right, while the bottom row shows agar phantom (II) with an inverted structure.

When moderate down sampling was applied, the reconstructed velocity maps in Figure 3C,D preserved nearly identical phase velocity distributions compared with the fully sampled results in Figure 3A,B. Although fine microstructural details were partially lost due to the omission of intermediate sampling points, the overall mechanical contrast and boundary locations remained clearly identifiable. In contrast, the low‐velocity region corresponding to the 1% agar exhibited substantial deviations from the fully sampled reference in Figure 3F. This degradation is attributed to the loss of spatial information beyond the resolvable limit and the subsequent linear interpolation process, which amplified noise contributions and propagated local estimation errors into the reconstructed velocity field.

Quantitative comparisons of phase velocity values for each homogeneous agar region are summarized in Figure 4. For both agar phantoms (I) and (II), the phase velocities obtained with down sampling (*N*_gap_ = 9) showed minimal differences; however, when the sampling gap exceeded the resolution threshold (*N*_gap_ = 69), the estimated phase velocity in the 1% agar region exhibited an abnormally large standard deviation (2.52 ± 2.96 m/s). An increase in phase velocity in MAE is observed with increasing *N*_gap_, reaching a maximum value of 0.3 m/s at *N*_gap_ = 69. These results indicate that excessive down sampling primarily affects spatial fidelity and boundary accuracy.

**FIGURE 4:**
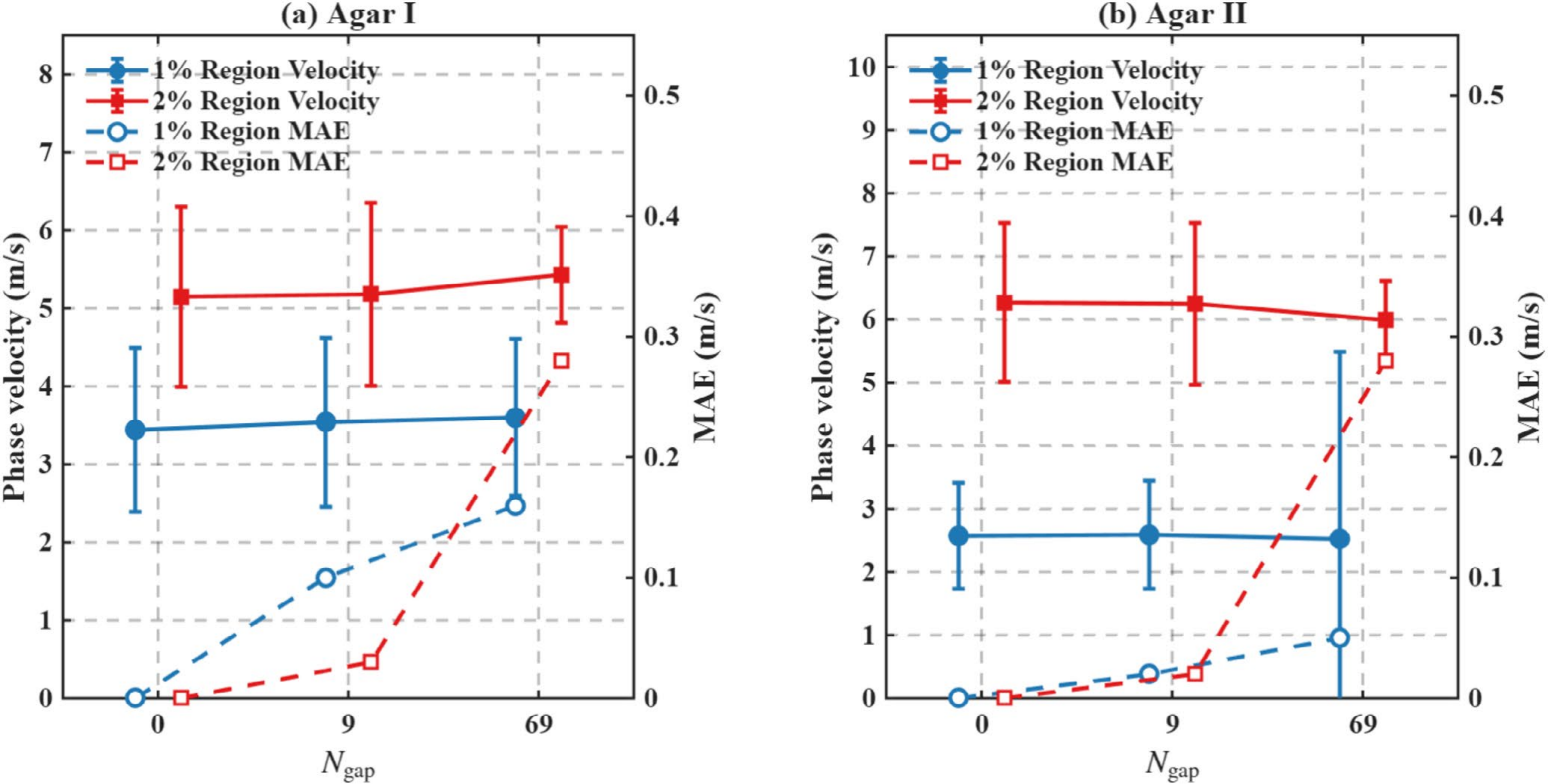
Phase velocity statistics of left and right agar regions under different down‐sampling conditions. MAE denotes the mean absolute error in mean phase velocity relative to the fully sampled case (*N*_gap_ = 0).

#### 5.1.2 Influence of Down Sampling on $R_{\mathrm{OCE}}$

To evaluate the influence of down sampling on $R_{\mathrm{OCE}}$ the λ distributions in the 1% and 2% agar regions, they were analysed using the fully sampled data. The smaller of the two regional average minimums λ was used as a conservative metric to quantify the capability of the system to resolve the smallest detectable mechanical features. For the fully sampled cases in Table 1, the averages λ were measured to be 1.47 mm for Agar (I) and 1.52 mm for agar (II) in the 1% region, respectively. Under moderate down‐sampling conditions (*W* = 10, *N*_gap_ = 9), these λ remained larger than the $R_{\mathrm{OCE}}$ (1.18 mm). As a result, the reconstructed phase velocity maps preserved accurate mechanical contrast and spatial continuity.

**TABLE 1:** Summary of λ measurements in homogeneous agar regions.

Phantom	Region	Wavelength (mean ± std, mm)
Agar I	1%	1.47 ± 0.36
	2%	2.42 ± 0.26
Agar II	1%	1.52 ± 0.36
	2%	2.36 ± 0.74

In contrast, when an excessively down sampling was used (*W* = 70, *N*_gap_ = 69), the λ became shorter than the minimum resolvable limit of the $R_{\mathrm{OCE}}$ (57 mm). Under this condition, the spatial sampling density was insufficient to support reliable phase‐gradient estimation, leading to degraded spatial fidelity and distorted velocity distributions. These results indicate that appropriate selection of both the down‐sampling interval and the phase‐gradient window size is critical for accurate phase velocity reconstruction.

### 5.2 3D OCE Results

#### 5.2.1 Influence of Sampling Density on Velocity Estimation

The 3D inclusion phantom consisted of a 1.5% agar base with a 2.5% injected inclusion and corresponding OCT frames are shown in Figure 5A,B. The estimated SAW velocity was approximately 9 m/s within the inclusion region, while the surrounding base exhibited velocities around 6 m/s. The Figure 5C,E,G present the MIP along the *Z* direction for volumetric datasets acquired at resolutions of 25 × 25, 50 × 50 and 100 × 100, respectively. At the lowest resolution, the elastography map shows limited spatial detail. The inclusion boundary is poorly defined and the velocity contrast between the inclusion region and the base is difficult to distinguish from noise. With increasing effective resolution, the inclusion structure becomes progressively clearer and the velocity distribution is more distinctly separated from the base region. The highest resolution dataset demonstrates improved boundary delineation and finer velocity variation within the inclusion region.

**FIGURE 5:**
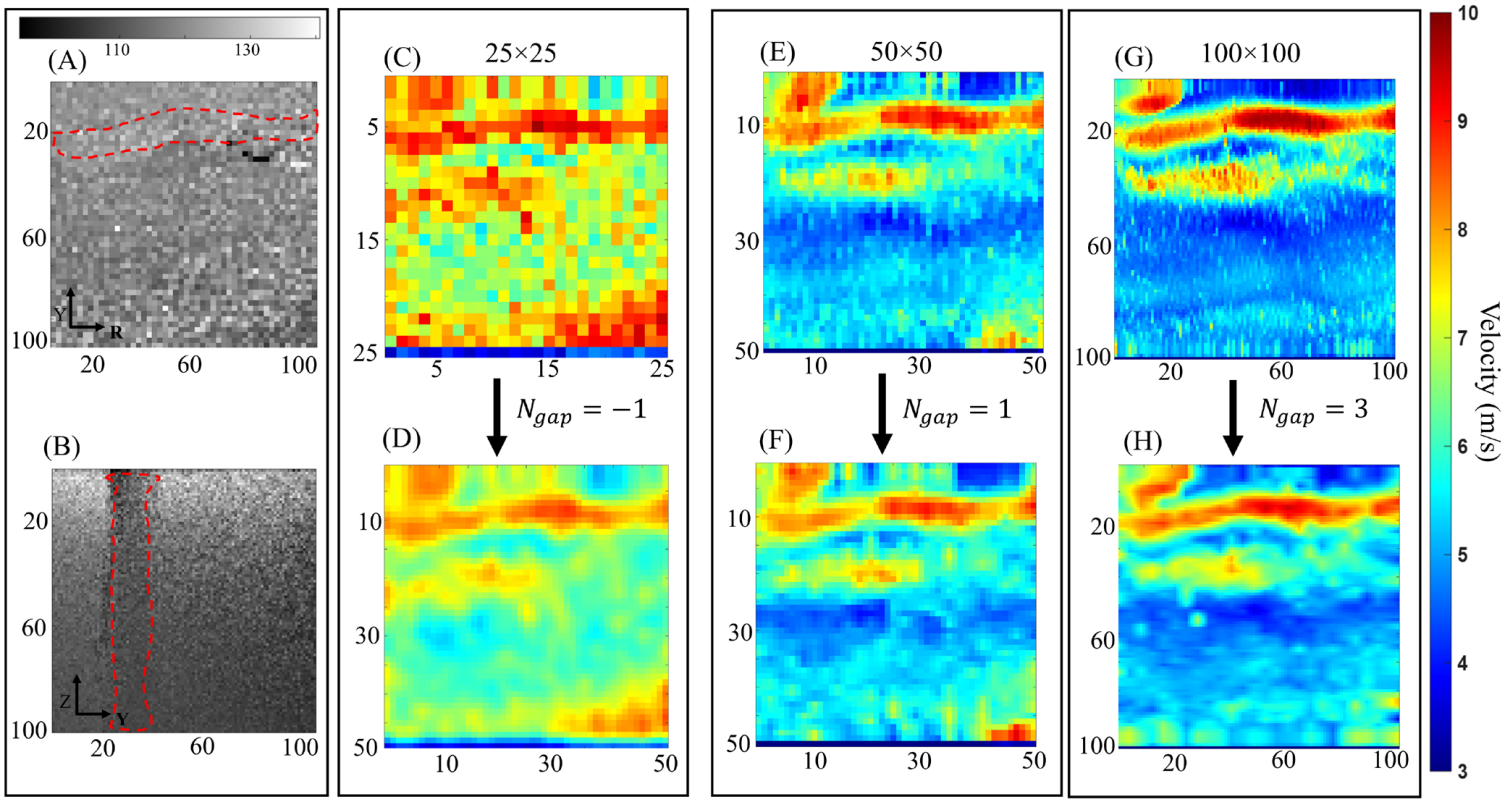
The MIP along the depth direction for the 3D inclusion phantom is presented. (A, B) OCT frames. The mechanical stimulator is positioned at the top of the image, generating wave propagation along the *X* direction.

To evaluate the effect of sampling density, cooperative up sampling results of Figure 5D and down sampling results of Figure 5F,H were performed and reconstructed after filling the missing points using spatial interpolation with *N*_gap_ values of −1, 1 and 3, respectively. The reconstructed maps preserve the overall inclusion morphology and velocity contrast, demonstrating improved boundary delineation and enhanced distinguishability between the inclusion and base regions. However, relative to the fully sampled HR reconstruction, fine structural details along the inclusion boundary appear partially blurred and a slight reduction in the maximum velocity value is observed (Figure 5F).

The phase velocity values are summarized in Figure 6. The MAE of average velocity between the fully sampled and sampled cases is below 0.4 m/s for both the 1.5% and 2.5% agar regions. However, when comparing Figure 6C with Figure 6E,G, a noticeable difference of approximately 2 m/s is observed in the 1.5% region. The sampling density in the 25 × 25 dataset limits the ability of the OCE map to effectively separate noise from valid wave signals, further contributing to the overestimation of velocity in this region.

**FIGURE 6:**
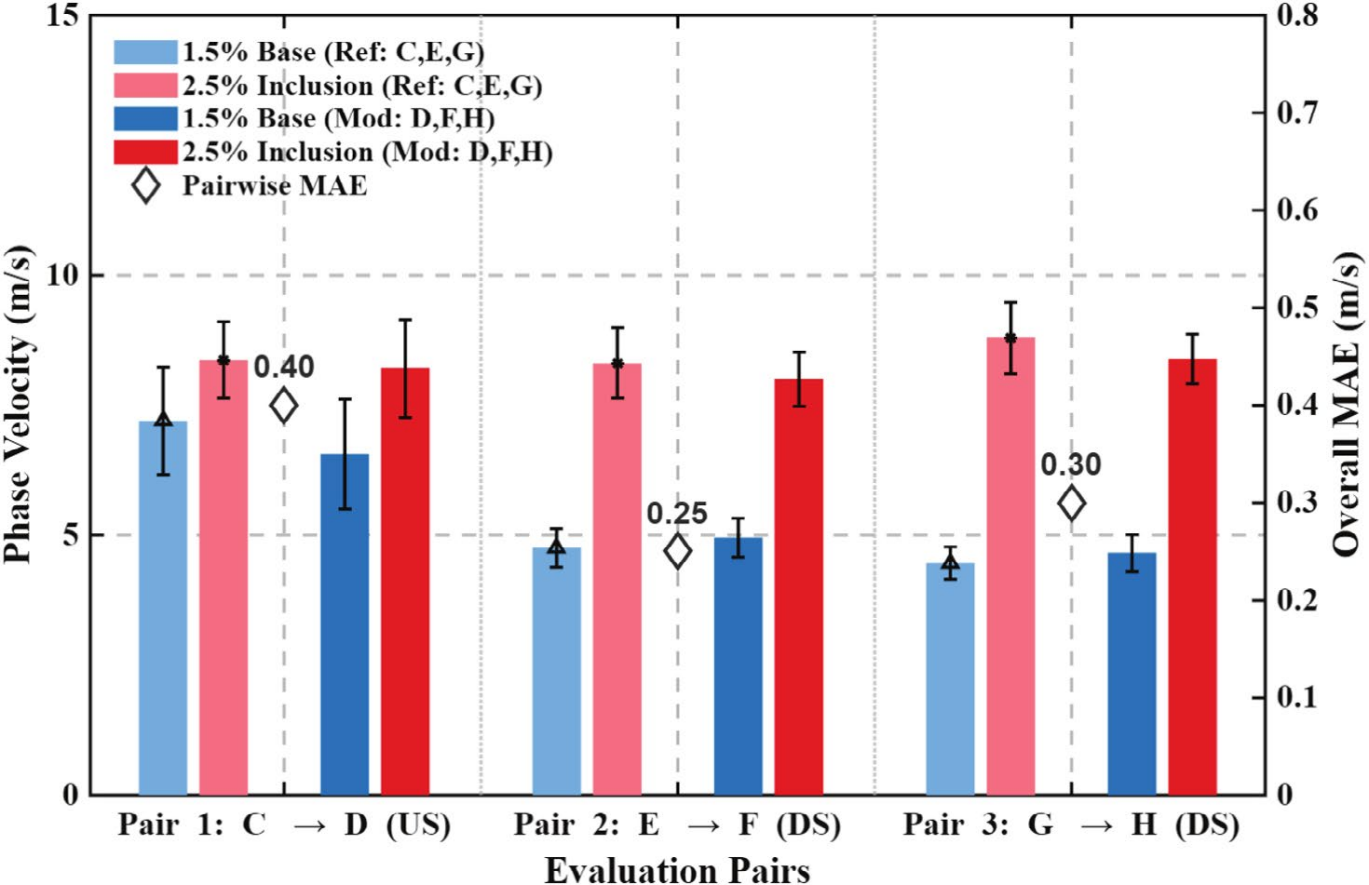
Phase velocity estimation in inclusion (2.5%) and base (1.5%). DS, down sampling; US, up sampling.

The CNR was used to quantify the mechanical contrast between the 2.5% agar inclusion and the 1.5% agar background in Figure 7. The CNR values of Figure 7D,F,H were calculated before interpolation. As summarized in Figure 7, both up sampling and down sampling preserved the CNR level across different sampling densities. The highest resolution case in (G) achieved improved contrast (2.4), while the corresponding downscaled result in (H) maintained a comparable CNR (2.75).

**FIGURE 7:**
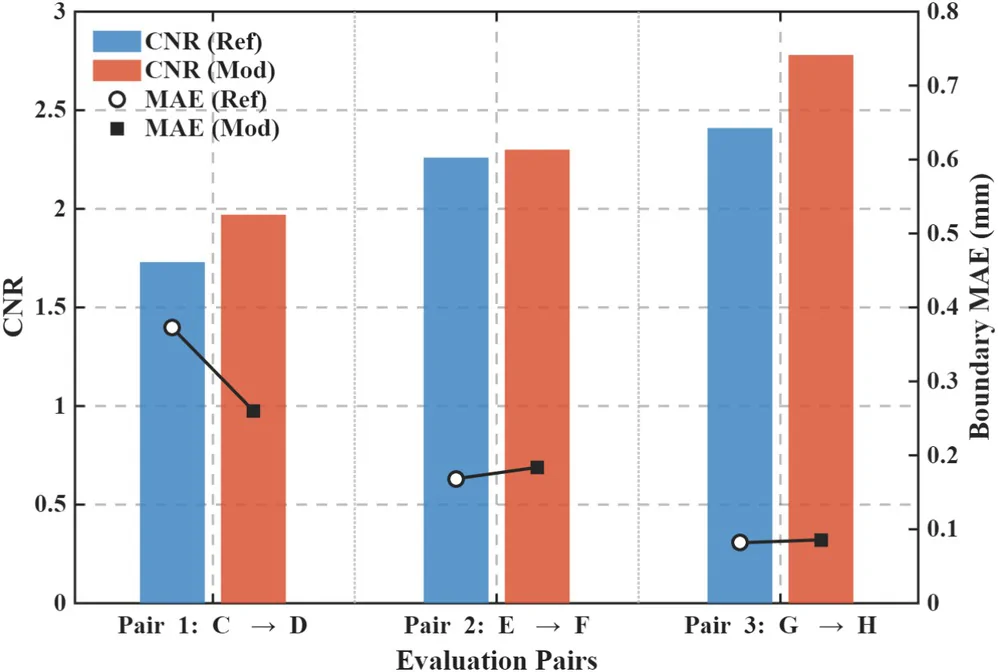
Quantitative evaluation of reconstructed elastogram using CNR and boundary localization MAE. DS, down sampling; US, up sampling.

Boundary localization accuracy was quantified using the MAE in Figure 7. The lowest resolution case (Figure 7C) exhibited the largest boundary offset (0.37 mm), whereas the corresponding up‐sampled result (Figure 7D) reduced the MAE to 0.26 mm. As the sampling resolution increased, the boundary offset progressively decreased and the corresponding down‐sampled results maintained comparable localization accuracy.

#### 5.2.2 Influence of Down Sampling on $R_{\mathrm{OCE}}$

The λ distribution along nY was calculated from the dominant frequency in Figure 8. The inclusion region exhibits a longer λ (3.83 ± 0.62 mm) compared with the base region (2.98 ± 0.38 mm), which is consistent with the higher phase velocity observed in the stiffer inclusion. To determine whether the sampling density affects the minimum spatial resolution constraint of OCE by Equation (5), the average λ in the base region (2.98 mm) was adopted as the conservative criterion. When the *N*_gap_ = −1 and *N*_gap_ = 1 with a sliding *W* of 3, the $R_{\mathrm{OCE}}$ is 0.44 mm. Besides, the $R_{\mathrm{OCE}}$ under *N*_gap_ = 3 and a sliding *W* size of 5 were calculated to be 1.43 mm. Therefore, the up sampling and down sampling configuration does not exceed the theoretical resolution constraint and remains sufficient to distinguish the inclusion from the base region.

**FIGURE 8:**
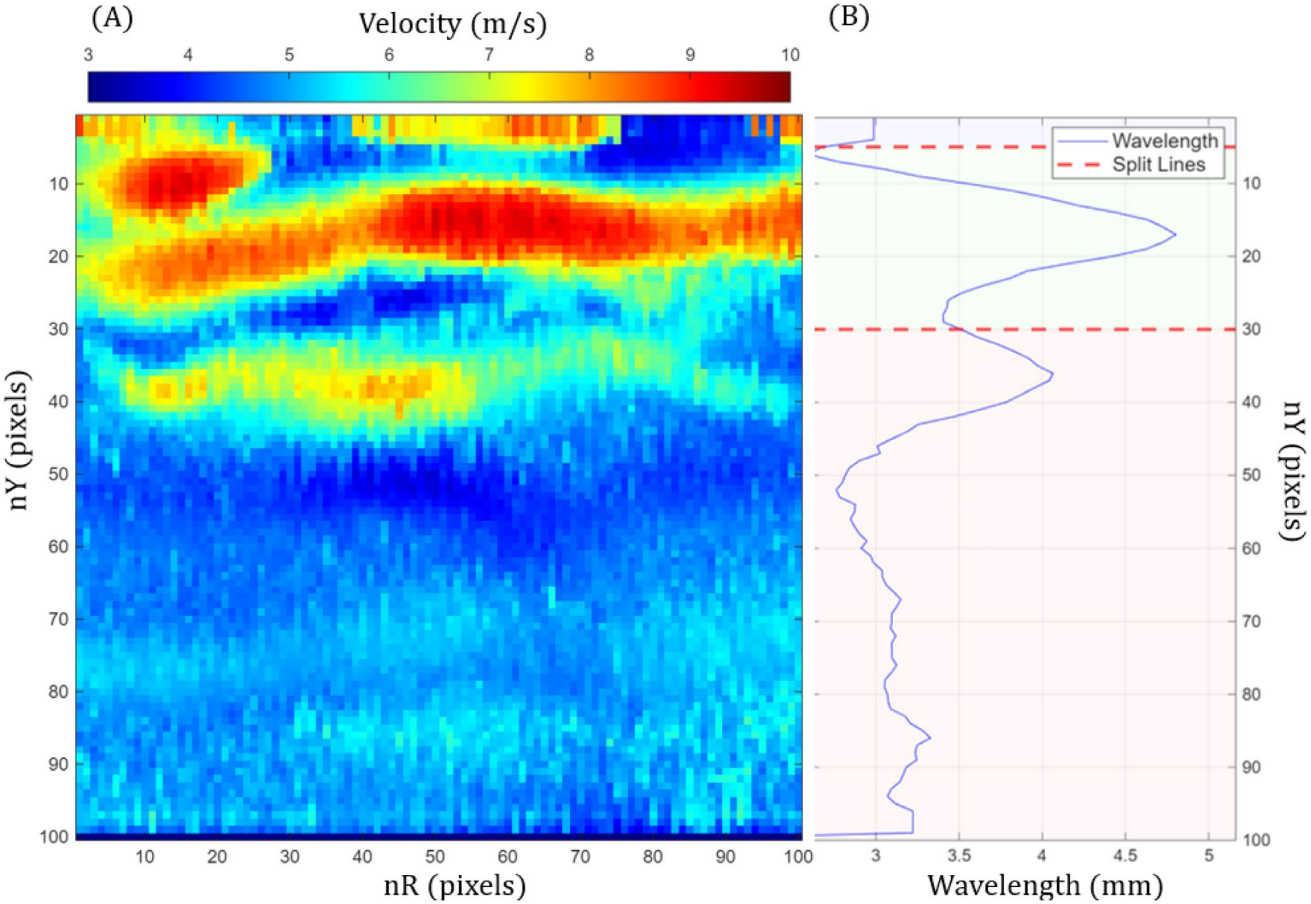
(A) Depth‐MIP elastogram reconstructed using a 100 × 100 sampling grid. (B) Spatial variation of the estimated wavelength along the nY direction.

#### 5.2.3 Results From Downstream Sampling Applied to Acne

The down‐sampling strategy was further applied to the human facial acne dataset. The structural OCT image is shown in Figure 9A, while the corresponding phase velocity map obtained using full sampling is presented in Figure 9B. The down‐sampled result with *N*_gap_ = 3 is shown in Figure 9C. Quantitative analysis showed that the estimated phase velocity changed by only 5.82% in the infected region and 2.25% in the normal region compared with the fully sampled result. In addition, the CNR decreased by only 8.28%, indicating that the mechanical contrast between the acne lesion and the surrounding normal tissue was well preserved after down sampling. The minimum λ was 2.78 ± 0.89 mm in the normal region and 4.66 ± 0.28 mm in the infected region, both of which are substantially larger than the minimum required λ (1.43 mm) predicted by Equation (5) for a sliding W size of 5. These results demonstrate that the sampling strategy satisfies the derived sampling criterion while maintaining reliable phase velocity estimation in practical in vivo imaging.

**FIGURE 9:**
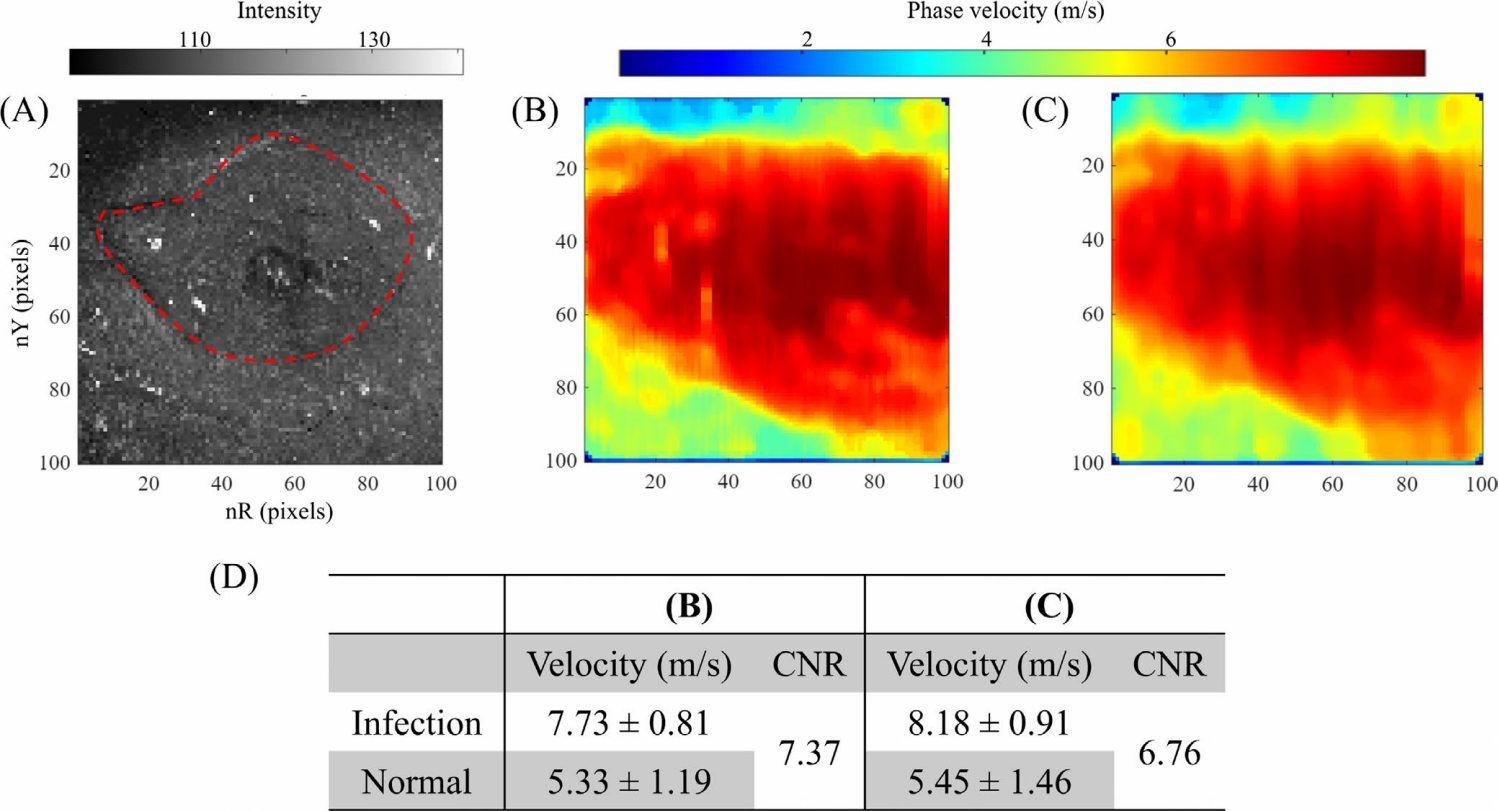
The MIP along the depth direction for the 3D human facial acne (red dashed circle). (A) OCT frames. (B) Fully sampled result. (C) Corresponding down‐sampling result. (D) Quantitative matrix comparison.

#### 5.2.4 The Computational Efficiency of Varied Sampling Density

The proposed algorithm was implemented in MATLAB (R2025b, MathWorks, Natick, MA, USA) and executed on a desktop computer equipped with an Intel Core i9‐13900K CPU and 64 GB RAM. The computational time was measured using MATLAB's built‐in time consumption function and the reported processing time includes (1) OCT data file opening and header reading; (2) spectral‐domain OCT reconstruction, including background subtraction, spectral windowing, Fourier transformation and conversion to OCT intensity images; (3) OCT surface detection based on an intensity threshold; (4) complex OCT data flattening and phase‐difference calculation between consecutive temporal frames; (5) phase‐gradient velocity estimation and (6) post‐processing of the velocity map, including missing value interpolation, median filtering and restoration to the original unflatten geometry.

The computational cost increased substantially with sampling density in Table 2. For fully scanned data, the processing time was 16.6 s for (C), 70.3 s for (E) and 309.3 s for (G). By comparison, the up‐sampled dataset (D) required 16.6 s, while the down‐sampled datasets (F) and (H) required 21.8 and 32.9 s, respectively. Overall, the reconstructed datasets based on up sampling or down sampling showed markedly lower computational cost than directly acquired datasets.

**TABLE 2:** Computational cost and relative improvement for different sampling resolutions.

Cases	Computational cost (s)	Improvement (%)
C (25 × 25)	16.6	—
E (50 × 50)	70.3	—
G (100 × 100)	309.3	—
D (US)	16.6	0
F (DS)	21.8	69
H (DS)	32.9	89.4

Abbreviations: DS, down sampling; US, up sampling.

## 6 Discussion

This study investigated the effect of spatial sampling density on OCE reconstruction and identified a trade‐off between spatial resolution, computational efficiency and velocity estimation stability. Practical guidelines for selecting the spatial sampling density and phase‐gradient estimation window size were established in Equation (5), ensuring adequate sampling of the SAW wavelength in the reconstructed OCE maps. The up‐sampling and down‐sampling strategies were quantitatively evaluated in agar inclusion phantoms using phase velocity error, CNR and boundary localization error. The results demonstrated that the approach maintained elastography accuracy while substantially reducing the post‐processing computational burden, particularly for HR OCE reconstruction.

The phase‐gradient velocity estimation method enables frequency‐specific velocity estimation and provides spatially resolved phase velocity mapping. By utilizing continuous spatial phase information, the method is well suited for characterizing harmonic excitations and frequency‐dependent mechanical waves. This feature is particularly advantageous for heterogeneous and dispersive materials, where local mechanical properties may vary spatially and exhibit frequency‐dependent behaviour [28].

However, there are some limitations in this paper. First, independent mechanical testing was not performed to validate the absolute mechanical properties of the samples. As the primary objective of this study was to investigate the influence of spatial sampling density on OCE reconstruction, the highest resolution acquisition was used as the reference for evaluating phase velocity consistency and boundary localization. Future studies incorporating independent mechanical characterization, such as compression testing, could further validate the quantitative accuracy of the proposed approach [29]. Nevertheless, structural continuity may be compromised by both up sampling and down sampling along the fast and slow scanning axes. Therefore, if structural integrity is the primary objective, sufficient A‐line sampling density should be maintained to avoid losing the mini target in the structure map. For the phase‐gradient velocity estimation, accurate phase unwrapping and local linear fitting within a sliding window are required to reconstruct reliable phase velocity. In regions with low SNR, phase discontinuities potentially reduce the stability of k estimation, which can be alleviated by optimized window selection and noise suppression during phase‐gradient processing. Finally, a fixed sampling interval may not be optimal across mechanically heterogeneous tissues. Because the mechanical wavelength depends on the local shear‐wave velocity at a given excitation frequency, regions with different mechanical properties may exhibit different spatial λ. Consequently, a sampling interval that adequately resolves the wave in one region may be insufficient in another, resulting in spatially varying effective resolution even under identical acquisition conditions [30]. Adaptive sampling strategies may therefore provide a more robust approach across heterogeneous tissues.

In this study, an acceptable trade‐off refers to maintaining the overall phase velocity distribution and mechanical contrast while limiting boundary localization error when the primary objective is to characterize relatively large mechanical features. Therefore, the sampling density should be selected according to the minimum mechanical λ, $R_{\mathrm{OCT}}$ and minimum feature size of interest. As a practical guideline, *W* = 3 is recommended to minimize spatial resolution loss, while *N*_gap_ = 1 can reduce the number of sampling points by approximately 50%. According to Equation (5), reliable phase velocity estimation can be maintained when the minimum SAW wavelength satisfies $\lambda >3.85\cdot R_{\mathrm{OCT}}$.

In clinical testing, reducing acquisition time is essential for improving patient comfort and minimizing operator‐dependent variability. Up sampling can improve the apparent spatial continuity of maps reconstructed from sparsely sampled data, potentially enabling reduced acquisition density without substantially compromising the visualization of large‐scale mechanical features, thereby promoting its application in dermatology [2] and ophthalmology [31]. In contrast, down‐sampling techniques can significantly reduce post‐processing time, which is particularly beneficial for systems with limited computational resources or in scenarios requiring rapid diagnostic feedback.

## 7 Conclusion

This study demonstrates that OCE acquisition and post‐processing efficiency can be substantially improved through appropriate spatial sampling and reconstruction strategies while maintaining reliable phase velocity mapping. A trade‐off criterion is derived from elastography resolution, integrating intrinsic OCT spatial resolution, excitation wavelength, sampling density and velocity estimation strategy, thereby providing a quantitative constraint for reliable boundary localization. The phase‐gradient velocity estimation method further enables frequency‐specific and spatially resolved estimation, supporting robust characterization of mechanical wave propagation. Overall, this work establishes an efficient sampling density optimization strategy for optical elastography, offering a practical solution to improve efficiency while preserving estimation accuracy.

## Funding

The authors have nothing to report.

## Conflicts of Interest

The authors declare no conflicts of interest.

## Data Availability

The data that support the findings of this study are available from the corresponding author upon reasonable request.
